# Genome sequences of actinobacteriophages JorRay, Blocker23, Nibbles, and OlgasClover

**DOI:** 10.1128/mra.01256-23

**Published:** 2024-03-06

**Authors:** Breanna M. Baumgartner, Kayla A. Bono, Dawn R. McIntosh, Anna M. Vu, Chanel F. Adams, Brooklyn C. Benik, Jessica Chavez, Samantha J. Gresky, Andres Sotelo, Jordan I. Ray, Alexandra Peister, Kendra W. Kimberley, Chelsey C. McKenna, James R. Theoret, Earl J. Yoon, Erin J. Windsor

**Affiliations:** 1 Department of Biological Sciences, College of Southern Nevada, Las Vegas, Nevada, USA; 2 Department of Biology, Morehouse College, Atlanta, Georgia, USA; University of Southern California, Los Angeles, California, USA

**Keywords:** bacteriophage, *Actinobacteria*, genome

## Abstract

JorRay, Blocker23, Nibbles, and OlgasClover are actinobacteriophages belonging to clusters G1, B2, CT, and DJ, respectively. JorRay and Blocker23 were identified in host bacterium *Mycobacterium smegmatis mc^2^155*. Nibbles and OlgasClover were identified in host bacterium *Gordonia rubripertincta* NRRL B-16540.

## ANNOUNCEMENT

*Actinobacteria* is a diverse phylum of Gram-positive bacteria, which includes pathogenic members, such as the *Mycobacterium* genus, and others that may be useful for environmental remediation like the *Gordonia* genus (1, 2). Actinobacteriophages are viruses that infect bacterial hosts belonging to the *Actinobacteria* phylum (1). Four actinobacteriophages, JorRay, Blocker23, Nibbles, and OlgasClover, were studied as part of the Science Education Alliance-Phage Hunters Advancing Genomics and Evolutionary Science (SEA-PHAGES) program (3).

Protocols for isolation of the bacteriophages and DNA extractions are from the SEA-PHAGES Phage Discovery Manual (4). Host bacterial strains were provided by the University of Pittsburgh via SEA-PHAGES. Soil samples were obtained by digging a few centimeters below the surface. Samples were incubated with PYCa (peptone-yeast-calcium) broth for 4 hours at 30°C and allowed to settle, and the supernatant was sterilized with a 0.22-µm filter. For direct isolation, supernatant was used immediately for plaque assays. For enriched isolation, 500 µL of the appropriate host bacteria was incubated with the supernatant at 30°C for 72 hours, 0.22-µm filter sterilized, and used for plaque assays. DNA was extracted from high-titer lysates using the Norgen phage DNA isolation kit with five rounds of freeze/thaw (4-minute freeze in dry ice-ethanol bath and 1-minute thaw).

Bacteriophages were sequenced at the Pittsburgh Bacteriophage Institute using an Illumina MiSeq instrument. Sequencing libraries were generated from extracted genomic DNA using Biolabs (NEB) NEB Ultra II Library Kit, v3, 150-base single-end reads, per the manufacturer’s instructions. The raw reads were uploaded to Newbler v.2.9 with default settings to produce contiguous assemblies of reads (5). The contigs produced from Newbler were analyzed using the default settings of Consed v.29 (http://www.phrap.org/consed/consed.html) to produce a single contig. Quality control included evaluating for completeness by checking genome circularization and accuracy by checking for gaps and low census quality, correcting errors, and determining genomic termini by searching for overrepresented portions of the DNA (5). JorRay was sequenced using the deconvolution of genomes after *en masse* sequencing (DOGEMS) method (6). For DOGEMS, genomic DNA of four different phages (including JorRay) were pooled into one library. This library was prepared, sequenced, and evaluated for quality in the same manner described earlier, resulting in contigs representing these four phages. JorRay was identified from the list of contigs by PCR using primers specific to G cluster phages (listed in Table 1). Genomic and sequencing information for bacteriophages is listed in Table 1.

**TABLE 1:** Actinobacteriophage Genome and Sequencing Information

Phage	GenBank accession number	SRA accession number	GPS coordinates	Isolation	Cluster	Genome length	GC content	Genome end type	Sequencing coverage	Number of reads	Primer sequences for DOGEMS
JorRay	ON637765	SRR23878859	33.75 N, 84.41 W	Enriched	G1	41,901 bp	66.6%	3′ sticky overhang (11 bases)	43	60,798	Forward: TCGACGTTCCGGGTACCTAT Reverse: TGACTCGGCCGATTTCGTAG
Blocker23	ON970593	SRR23878846	33.7488 N, 84.4153 W	Enriched	B2	67,402 bp	68.9%	Circularly permuted	1322	626,379	N/A
Nibbles	OR253918	SRR23878852	36.1789 N, 115.1841 W	Enriched	CT	45,601 bp	61.7%	3′ sticky overhang (10 bases)	2696	865,906	N/A
OlgasClover	OR613475	SRR23878849	36.1253 N, 115.2774 W	Direct	DJ	61,926 bp	51.3%	3′ sticky overhang (nine bases)	823	354,055	N/A

Annotations were done using the following programs: DNA Master v5.23.2 (http://cobamide2.bio.pitt.edu/computer.htm), Starterator v1.2 (https://github.com/SEA-PHAGES/starterator), Phamerator (phamerator.org) (7), PhagesDB BLAST (phagesdb.org/blast) (8), NCBI BLAST (9), PECAAN (discover.kbrinsgd.org), GeneMark v2.5p (10), Glimmer 3.02 (11), Aragorn (v1.1 and v1.2.38) (12), HHPRED (v3.2.0) (13), tRNAscanSE 2.0 (14), TMHMM (v2.0) (15), and SOSUI (v1.11) (16) using the default parameters listed in the SEA-PHAGES bioinformatic guide (17).

JorRay contains 62 forward-facing genes except for tyrosine integrase and immunity repressor being reverse, a hallmark of the G1 cluster. Blocker23 contains 91 genes, and 31 were assigned putative functions. The genome begins with genes predicted to belong to the PreQ_0_ pathway of 7-deazaguanine modifications protecting bacteriophage DNA from host restriction enzymes (18), which were not found in the other phages. Nibbles contains 67 genes. Gene 55 is a putative lipoprotein, identified in 4 of 47 cluster members (Azira, Fribs8, and Survivors). OlgasClover contains 93 forward-facing genes, and 25 genes were assigned putative functions. No genes encoding transfer RNAs or transfer-messenger RNAs were found in these phages.

## Data Availability

The GenBank accession numbers and SRA accession numbers for the four bacteriophages reported here are in Table 1.
